# Supplementary material for: Genetically controlled mtDNA deletions prevent ROS damage by arresting oxidative phosphorylation
Source: eLife. 2022 Jul 8;11:e76095. doi: 10.7554/eLife.76095 (PMC9427111; doi:10.7554/eLife.76095)
Supplement: Supplementary file 1. [file elife-76095-supp1.docx]

**Supplementary File 1**

List of selection agents used in evolution experiments

| **Selection** | **Compound** | **Concentration** | **Type** |
| --- | --- | --- | --- |
| Mitochondrial $O_{2}^{-}$ stress | Paraquat | 400 µg/mL | Mitochondrial $O_{2}^{-}$ producer |
| Arsenic (III) stress | Arsenite (NaAsO_2_) | 3 mM | Toxic metalloid |
| Rapamycin stress | Sirolimus | 0.8 µg/mL | TOR-inhibitor |
| Citric Acid stress | Citric Acid | 62.5 mg/mL | Weak organic acid |
| Glycine use | L-Glycine | 160.86 mg/mL (30 mg N/mL) | Nitrogen source |
| Citrulline use | L-Citrulline | 125.14 mg/mL (30 mg N/mL) | Nitrogen source |
| Tryptophan use | L-Tryptophan | 218.82 mg/mL (30 mg N/mL) | Nitrogen source |
| Isoleucine use | L-Isoleucine | 281.1 mg/mL (30 mg N/mL) | Nitrogen source |
